# Inherited and Acquired Determinants of Hepatic CYP3A Activity in Humans

**DOI:** 10.3389/fgene.2020.00944

**Published:** 2020-08-21

**Authors:** Johannes Matthaei, Wagner Hugo Bonat, Reinhold Kerb, Mladen Vassilev Tzvetkov, Jakob Strube, Stefanie Brunke, Cordula Sachse-Seeboth, Daniel Sehrt, Ute Hofmann, Jacob von Bornemann Hjelmborg, Matthias Schwab, Jürgen Brockmöller

**Affiliations:** ^1^Institute for Clinical Pharmacology, University Medical Center Göttingen, Georg-August University, Göttingen, Germany; ^2^Department of Epidemiology, Biostatistics and Biodemography, University of Southern Denmark, Odense, Denmark; ^3^Dr. Margarete Fischer-Bosch Institute of Clinical Pharmacology and University of Tübingen, Stuttgart, Germany; ^4^Department of Clinical Pharmacology, University Hospital Tübingen, Tübingen, Germany; ^5^Department of Pharmacy and Biochemistry, University of Tübingen, Tübingen, Germany

**Keywords:** CYP3A, CYP3A4, CYP3A5, midazolam, 4ß-OH-cholesterol, 6ß-OH-cortisol, heritability, twin study

## Abstract

Human CYP3A enzymes (including CYP3A4 and CYP4A5) metabolize about 40% of all drugs and numerous other environmental and endogenous substances. CYP3A activity is highly variable within and between humans. As a consequence, therapy with standard doses often results in too low or too high blood and tissue concentrations resulting in therapeutic failure or dose-related adverse reactions. It is an unanswered question how much of the big interindividual variation in CYP3A activity is caused by genetic or by environmental factors. This question can be answered by the twin study approach. Using midazolam as CYP3A probe drug, we studied 43 monozygotic and 14 dizygotic twins and measured midazolam and its metabolite 1-OH-midazolam. In addition, endogenous biomarkers of CYP3A activity, 4ß-OH-cholesterol and 6ß-OH-cortisol, were analyzed. Additive genetic effects accounted for only 15% of the variation in midazolam AUC, whereas 48% was attributed to common environmental factors. In contrast, 73, 56, and 31% of 1-OH-midazolam, 4ß-OH-cholesterol and 6ß-OH-cortisol variation was due to genetic effects. There was a low phenotypic correlation between the four CYP3A biomarkers. Only between midazolam and its 1-OH-metabolite, and between midazolam and 6ß-OH-cortisol we found significant bivariate genetic correlations. Midazolam AUC differed depending on the *CYP3A4^∗^22* variant (*p* = 0.001) whereas plasma 4ß-OH-cholesterol was significantly lower in homozygous carriers of *CYP3A5^∗^3* (*p* = 0.02). Apparently, non-genomic factors played a dominant role in the inter-individual variation of the CYP3A probe drug midazolam. A small intra-individual pharmacokinetic variation after repeated administration of midazolam was rated earlier as indication of high heritability of CYP3A activity, but according to present data that could also largely be due to constant environmental factors and/or heritability of liver blood flow. The higher heritabilities of 4ß-OH-cholesterol and of 1-OH-midazolam may deserve further research on the underlying factors beyond CYP3A genes.

**Clinical Trial Registration:** ClinicalTrials.gov: NCT01845194 and EUDRA-CT: 2008-006223-31.

## Introduction

The cytochrome P450 (CYP) enzymes 3A4 and 3A5 play a key role in the biotransformation of about 40% of currently used drugs (Evans and Relling, 1999). CYP3A enzymes also catalyze the biotransformation of numerous exogenous and endogenous substances, notably cholesterol, bile acids, and many steroid hormones (Patki et al., 2003; Bodin et al., 2005; Klein and Zanger, 2013). CYP3A4 is the most prominent CYP3A subtype in adult humans. The homologous isozyme CYP3A5 has partially overlapping substrate specificity with CYP3A4 but may significantly contribute to CYP3A metabolic activity with some drugs like tacrolimus. Only about 10–15% of persons with European ancestry express CYP3A5 and that can be easily analyzed by genotyping. However, only a small fraction of the variation in CYP3A4 expression and activity can thus far be explained by defined genotypes.

Expression and activity of human CYP3A enzymes in the gut epithelia and liver cells is highly variable both inter- as well as intra-individually (Koch et al., 2002). CYP3A activity may be reduced to 10% by strong inhibitors like itraconazole, ritonavir, or grapefruit juice (Kim et al., 2006; Greenblatt and Harmatz, 2015). It could also be increased up to 10-fold by strong transcriptional inducers like rifampin, carbamazepin or phenobarbital (Bodin et al., 2001; Bui et al., 2016). However, even when excluding such strong environmental modulators of CYP3A activity and expression, there remains a very wide and mostly unexplained variation between individuals (Gorski et al., 1998; Gashaw et al., 2003). By extensive search for genetic polymorphisms in the CYP3A gene locus that could account for inter-individual variation in CYP3A activity only a few variants were found that explained overall only a small fraction of that variation (Klein and Zanger, 2013). Also, polymorphisms in nuclear transcription factors and other regulators of expression appear to contribute to the variation to a minor extent only. A better understanding of the inter-individual variation in CYP3A activity may result in molecular biomarkers that could facilitate individually more precise dosing of drugs metabolized by CYP3A.

Our understanding of CYP3A variation depends on how the CYP3A phenotype was measured. As human liver tissue or intestinal tissue is mostly not available in clinical research, *in vivo* probe drugs are the most important tools for the study of individual CYP3A activity. Numerous CYP3A probe drugs have been applied. Currently, midazolam is the most widely used (Thummel et al., 1994; Ozdemir et al., 2000; Tateishi et al., 2001; Eap et al., 2004a, b; Kirby et al., 2006; Fromm et al., 2007; Fuhr et al., 2007; Tomalik-Scharte et al., 2014), mostly because of its high sensitivity in reflecting even moderate enzyme inhibition and induction. Approximately 75% of midazolam is eliminated as 1-OH-midazolam (Heizmann et al., 1983), and this metabolite is formed by CYP3A (Kronbach et al., 1989). As an alternative to administering probe drugs, individual CYP3A activity could also be determined using endogenous biomarkers, such as 4ß-OH-cholesterol (Tomalik-Scharte et al., 2009; Diczfalusy et al., 2011) and 6ß-OH-cortisol (Galteau and Shamsa, 2003) that are generated by CYP3A-mediated oxidation of cholesterol and cortisol.

Upon repeated drug administration of CYP3A substrates, a very high intra-individual constancy was reported for midazolam (Ozdemir et al., 2000). From such data, it was interpreted that up to 90% of variation in human CYP3A activity is caused by genetic factors (Ozdemir et al., 2000). However, individual constancy may also be due to constant environmental effects or epigenetic factors.

An analysis of genetic factors determining enzyme induction by St. John’s wort showed that ca. 66% of the inter-individual variation in induced CYP3A4 activity (measured with quinine as probe drug) may be due to additive genetic factors (Rahmioglu et al., 2011). However, induced CYP3A activity is not only reflecting the heritable factors influencing the pharmacokinetics of the CYP3A probe drug, but also by those that affect pharmacokinetics of the inducer (Rahmioglu et al., 2011).

Thus, at present, it is not clear how much of the inter-individual variation in CYP3A4 activity is in fact genetically determined. Twin studies performed in monozygotic (MZ) and dizygotic (DZ) twins allow to distinguish between additive (A) and dominant (D) genetic factors on the one hand, and constant (C) and unique environmental (E) factors on the other hand. We used this approach with midazolam as CYP3A4 probe drug and endogenous biomarkers of CYP3A activity. To allow comparison with the repeated drug administration approach (Ozdemir et al., 2000), each CYP3A test was performed three times in each individual. In conclusion, this study should contribute to a better understanding of the inter-individual variation of human CYP3A activity, which plays a relevant role in up to 40% of current drug therapies as well as in the metabolism of many endogenous and exogenous substances with biomedical relevance.

## Materials and Methods

### Study Design

The study was a repeated dose pharmacokinetic/pharmaco- genetic study on monozygotic (MZ) and dizygotic (DZ) twin pairs. The ethics committee of the University of Göttingen and the German Federal Drug Administration (BfArM) approved the study. It was registered at ClinicalTrials.gov (NCT01845194) and in the European clinical trials database (EUDRA-CT number: 2008-006223-31).

In total, 116 subjects including 44 MZ and 14 same-sex DZ twin pairs were included in the study. One MZ twin pair was excluded from the analyses of the intravenous midazolam administration because of erroneous blood sampling. All subjects were healthy according to their medical history, medical examination, electrocardiogram, urine status, and clinical chemistry analyses. The latter included sodium, potassium, calcium, aspartate aminotransferase, creatinine, total bilirubin, hemoglobin, erythrocyte, thrombocyte, and leucocyte counts. Written informed consent was obtained from each subject before participation in the study. Except for hormonal contraceptives, no other drugs or potentially interacting nutrients (grapefruits, grapefruit juice, St. John’s wort) were allowed for 1 week prior to the study days and until the last blood and urine sampling was completed. Smoking was allowed if both siblings of each pair smoked to a similar extent. Subjects with history of alcohol or drug addiction were excluded prior to any study related procedures. Additionally, subjects were not allowed to drink alcohol from 48 h prior to 72 h after midazolam application and alcohol breath test was absolved in the morning of each study day. Other dietary habits were recorded in standardized nutrition questionnaires (Winkler and Doring, 1998). Lean body weight was calculated from a formula based on body weight and height (Hume, 1966).

Midazolam was administered as a 2 min intravenous infusion with a total dose of 0.2 mg. The phenotyping test with midazolam was performed on three different occasions with a time interval of at least 1 week and maximally 3 months. Blood samples were drawn at 0 (before infusion), 0.25, 0.5, 0.75, 1, 1.5, 2, 2.5, 3, 4, 5, 6, 7, 8, and 24 h after the midazolam infusion.

Cholesterol and 4ß-OH cholesterol were measured in the baseline blood samples before drug administration. All samples were centrifuged for 10 min (4000 rpm) at 4°C and the plasma was stored at −20°C. To avoid possible circadian variation of the cortisol to 6ß-OH-cortisol metabolism (Ohno et al., 2000), a 24 h urine sampling for quantification of cortisol and 6ß-OH cortisol was performed on each of the three study days.

### Bioanalytical Methods

The concentrations of midazolam, 1-OH-midazolam, 6-ß-hydroxycortisol and cortisol were determined by LC-MS-MS analysis on an Agilent 6460 triple quadrupole mass spectrometer (Agilent, Waldbronn, Germany). Sample work-up and LC-MS-MS analysis of midazolam was performed as described previously (Matthaei et al., 2015; Thiel et al., 2015) using midazolam-d6 (Roche Nr. RO0213981-010) as internal standard. For 1-OH-midazolam, 1 ml of plasma was extracted with diethyl ether as described (Becquemont et al., 2006) using 1-OH-midazolam-d5 (Toronto Research Chemicals, no. H948423) as internal standard. Mass spectrometric conditions and transitions in multiple reaction monitoring (MRM) mode were as described previously (Thiel et al., 2015). Standardization of the analytical assays was performed with calibration samples prepared in plasma in the concentration range from 0.04 to 20 nM for 1′-hydroxymidazolam, and 0.2–100 nM for midazolam. Precision of the analytical method at the limit of quantification, which was 0.04 nM for 1′-hydroxymidazolam and 0.2 nM for midazolam, could be characterized with a coefficient of variation (CV) of 6.6 and 3.7% and a bias of 6.1 or 1.5% for 1′-hydroxymidazolam and midazolam, respectively. At higher concentrations (0.1, 1, and 15 nM for 1′-hydroxymidazolam and 0.5, 5, and 75 nM for midazolam) the CV ranged from 1.3 to 8.1%, and the bias was between −3.8 and 4.2%.

Urine samples were centrifuged after thawing, and 10 μl were diluted with 90 μl of water for determination of cortisol and 6ß-OH-cortisol. After addition of internal standard mixture (10 pmol cortisol-d4 and 100 pmol 6ß-OH-cortisol-d4) the samples were centrifuged, and 10 μl of the supernatant were used for LC-MS-MS analysis. HPLC separation was achieved on a reversed phase column (Synergi^TM^ 4 μm Polar RP 80Å, 150 × 2 mm, Phenomenex, Aschaffenburg, Germany) with a gradient of acetonitrile and water with 0.1% formic acid.

Cholesterol and 4ß-hydroxycholesterol were determined by GC-MS in EI mode, on a 5975 XL MSD coupled to a 7890A GC (Agilent, Waldbronn, Germany). Cholesterol quantification was performed as described previously (Lutjohann et al., 1995) with minor modifications. Briefly, 10 μl of plasma were spiked with 10 μg of cholesterol-d5 as internal standard. After saponification with 0.5 ml of 1 M NaOH in 90% ethanol at 70°C for 1 h, 250 μl of water were added and the samples extracted with 2 ml of n-hexane. An aliquot of 50 μl of the extract was evaporated to dryness and derivatized with 20 μl of *N,O*-bis(trimethylsilyl)trifluoroacetamide (BSTFA) for 30 min at room temperature.

Sample preparation for 4ß-hydroxycholesterol was performed according to a previously published method (Dzeletovic et al., 1995) with minor modifications. Briefly, 1 ml of plasma was spiked with 10 μg of BHT and 50 ng of the internal standard 4ß-hydroxycholesterol-d4 and saponified with 2.5 ml of 1 M NaOH in 90% ethanol at 70°C for 1 h under argon and then extracted with 1 ml of water and 5 ml of chloroform. The chloroform phase was evaporated to dryness in a stream of nitrogen and the residue dissolved in 1 ml of toluene. Samples were purified by solid phase extraction on silica cartridges (Isolute Si 100 mg, 3 ml, Biotage, Uppsala, Sweden) preconditioned with hexane. The cartridges were washed with 1 ml of hexane and 10 ml of 2-propanol in hexane (0.5% v/v) and then eluted with 2 ml of 2-propanol in hexane (30% v/v). The eluate was evaporated to dryness in a stream of nitrogen and derivatized with 20 μl of BSTFA for 30 min at room temperature. GC was performed on a J&W DB-5MS column (25 m, 0.2 mm i.d., 0.33 μm film thickness; Agilent) in the splitless mode. For the analysis of cholesterol, the GC oven program started at 150°C and was held for 1 min. Temperature was increased with 20°C/min to 300°C, with a total run time of 18.5 min. The trimethylsilyl derivatives of cholesterol and the internal standard cholesterol-d5 were detected in SIM mode at m/z 458 and 463, respectively. For the analysis of 4ß-hydroxycholesterol the GC oven program startet at 150°C and was held for 1 min. Temperature was increased with 10°C/min to 250°C, then with 30°C/min to 300°C. The trimethylsilyl derivatives of 4ß-hydroxycholesterol and the internal standard 4ß-hydroxycholesterol-d4 were quantified in SIM mode at m/z 366 and 370, respectively using m/z 456 and 460 as qualifier ions.

Calibration samples for cholesterol were prepared in isooctane with 10% 2-propanol in the concentration range from 1 to 30 μg. Calibration samples for 4ß-hydroxycholesterol were prepared in isooctane with 0.8% 2-propanol from 5 to 100 ng. Calibration samples for cortisol and 6ß-hydroxycortisol were prepared in water from 0.025 to 10 pmol, or 0.25 to 100 pmol, respectively. Calibration samples were worked up as the samples, and analyzed together with the unknown samples. Calibration curves based on internal standard calibration were obtained by weighted (1/x) linear regression for the peak area ratio of the analyte to the respective internal standard against the amount of the analyte. The concentration in unknown samples was obtained from the regression line.

### Genotyping

DNA was isolated from whole venous blood by automated solid phase extraction with the EZ1^TM^ DNA Blood 350 μl Kit using the Bio-robot EZ1^TM^ (both Qiagen, Hilden, Germany). The *CYP3A4^∗^1b* and *^∗^22* alleles as well as the *CYP3A5^∗^1/^∗^3* polymorphism were genotyped by DNA sequencing using fluorescence-labeled dideoxynucleotides and detection via Gene Mapper v3.7 Software^®^ (Applied Biosystems^®^, Foster City, United States). Zygosity was assessed by analyzing variants in 23 polymorphic genes. Monozygosity was concluded when there were no differences regarding the polymorphisms in both siblings of one twin pair.

### Pharmacokinetic Analysis

Pharmacokinetic parameters were estimated by non-compartmental analyses using the WinNonlin software (Pharsight Corporation, Mountain View, United States). The AUC from zero to infinity (AUC), was calculated by the linear/log trapezoidal rule and using the terminal elimination rate constant (lambda z). AUC_420_ was calculated from time of dose to 420 min after dosage. Total plasma clearance (Cl) after administration of midazolam was calculated as the ratio dose over AUC. Time (*t*_max_) and amount of maximum plasma concentration (C_max_) were given as measured and the terminal half-life (*t*_1/2_) was calculated as *t*_1/2_ = ln(2)/lambda z.

### Statistics and Analysis of Heritability

Predefined primary endpoint was midazolam AUC. All concentrations and pharmacokinetic parameters were determined on three independent occasions to compare intra- versus interindividual variation. The genetic component (rGC) concerning intra- (SD_W_) and inter-individual (SD_B_) standard deviation was calculated as described by Kalow et al. (1998, 1999). In structural equation modeling of heritability and in the other multifactorial analyses the individual means from the measurements or parameters obtained on the three occasions were used. Using structural equation analysis, variation was separated into additive genetic (A), common environmental (C), and unique environmental (E) effects. The heritability is the proportion of the variance resulting from the genetic effect (A). In order to investigate the genetic, common environmental, and unique environmental effects of the different traits taken into account, the possible correlation between traits were fitted in multivariate ACE, AE, and CE biometrical models using the mcglm package for multivariate regression analysis in R. As an exploratory analysis, we computed the correlation between MZ and DZ twins based on an unstructured (equal mean and variance for MZ and DZ twins) model. In all models, the mean structure for each trait was specified as a linear function of the covariates age and sex.

## Results

We studied 44 monozygotic and 14 dizygotic twin pairs. There were no significant differences in demographic data between MZ and DZ twin pairs (Table 1). No serious adverse events related to the drugs or procedures of this study were observed.

**TABLE 1:** Demographic data of study participants.

	Total	Sex		Age	Weight	Height
		Female	Male	[years]	[kg]	[m]
				
	*n*	*n*	*n*	Mean		
		
	(% of each group)			(Range)		
Monozygotic	88 (75.9)	54 (61.4)	34 (38.6)	25.8 (18 – 56)	66.7 (48.0 – 97.5)	1.72 (1.55 – 1.95)
Dizygotic	28 (24.1)	20 (71.4)	8 (28.6)	23.1 (18 – 36)	67.0 (53.5 – 83.5)	1.71 (1.60 – 1.95)

**FIGURE 1:**
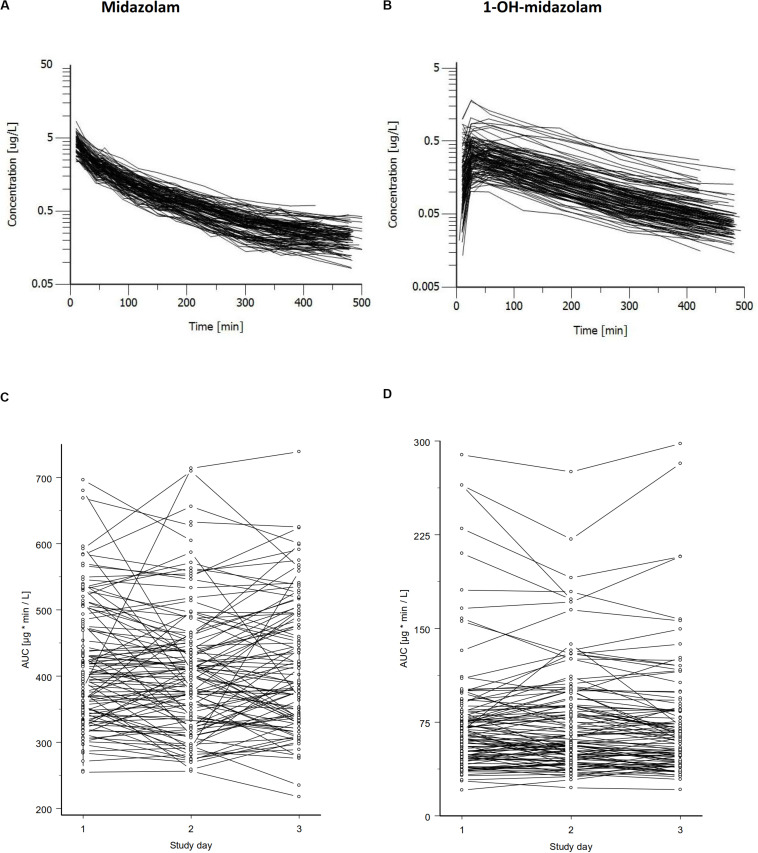
Concentration time curves of midazolam **(A)** and its major metabolite 1-OH-midazolam **(B)** after intravenous administration of 0.2 mg midazolam. Data for all subjects of one study day are shown. Concentrations of midazolam varied about 10-fold and that of the CYP3A dependent metabolite even higher. Midazolam AUC **(C)** and 1-OH-midazolam AUC **(D)** is shown below illustrating the intra-individual variation and the variation between the three study days. The genetic component (Kalow et al., 1998) was determined from this data independently of the twin status, with values of 0.57 (95% CI = 0.54 – 0.60) for the AUC of midazolam and 0.93 (95% CI = 0.93 – 0.94) for the AUC of 1-OH-midazolam.

Midazolam was administered intravenously on three independent occasions under supine and resting conditions. As illustrated in Figure 1, there was a significant variation in midazolam blood concentrations between the individuals and an even bigger variation in 1-OH-midazolam blood concentrations. Inter-individually, there was a 3.1-fold variation in midazolam AUC (288 – 897.4 μg^∗^min/L) and a 10.4-fold variation of 1-OH-midazolam AUC (30.95 – 322.8). There were no statistically significant differences in the pharmacokinetic parameters between the monozygotic and dizygotic twins (Table 2). Intra-individually (i.e., between-days), the relative variation was similar for midazolam and for 1-OH-midazolam with mean coefficients of variation of 11.3 and 12.3%.

**TABLE 2:** Pharmacokinetic parameters of midazolam and 1-OH-midazolam.

	MZ	DZ
**Midazolam**		
AUC [μg * min/L]	450.3 (290.3 – 897.4)	431.0 (287.8 – 640.7)
AUC_420_ [μg * min/L]	378.6 (255.8 – 636.4)	354.8 (240.0 – 542.9)
Cl [L/min]	0.45 (0.23 – 0.69)	0.48 (0.32 – 0.70)
Cl [ml/min/kg]	6.80 (3.02 – 11.80)	6.74 (4.94 – 10.27)
*t*_1/2_ [min]	163 (97 – 552)	185 (102 – 505)
C_max_ [μg/L]	4.11 (2.71 – 9.00)	3.76 (2.34 – 6.88)
*t*_max_ [min]	10 (9 – 20)	10 (10 – 19)
**1-OH-midazolam**		
AUC [μg * min/L]	66.88 (33.75 – 195.6)	73.31 (30.95 – 322.8)
Ratio 1-OH-midazolam/midazolam (AUC)	0.15 (0.09 – 0.61)	0.17 (0.07 – 0.54)

*Data is given in median (minimum – maximum). AUC, Area under the concentration time curve extrapolated to infinity; AUC_420_, Area under the concentration time curve from 0 to 420 min after midazolam administration; Cl, total body clearance; *t*_1/2_, terminal elimination half-life; C_max_, maximum concentration; *t*_max_, time of maximum concentration.*

As illustrated in the lower part of Figure 1, in some, but not in all volunteers a high intraindividual constancy of midazolam and 1-OH-midazolam AUC was found. From comparing the intra- and interindividual variation, the so-called genetic component was calculated. The genetic component is an alternative approach suggested as an indicator of heritability (Kalow et al., 1998, 1999). This parameter is determined completely independently of the twin status and solely based on intra- and inter-individual variability. The genetic component was 0.612 for midazolam and 0.931 for 1-OH-midazolam (Table 3). That means, that up to 61% of the variation in midazolam and up to 93% of the variation in 1-OH-midazolam may be due to genetic factors. This parameter was also calculated for the endogenous biomarkers plasma 4ß-OH-cholesterol and 24-h urinary 6ß-OH-cortisol with a quite high genetic component of 0.812 for the cholesterol metabolite. However, in interpretation of this high value it has to be taken into account that 4ß-OH-cholesterol has a long half-life of more than 60 h (Bodin et al., 2002), and thus some of the individual constancy is simply due to that. As illustrated in Table 4, there were no significant differences in the endogenous CYP3A biomarkers between monozygotic and dizygotic twins.

**TABLE 3:** Within- and between subject variance and genetic component of the 4 biomarkers.

	Midazolam AUC	1-OH-midazolam AUC	4ß-OH-cholesterol Plasma concentration	6ß-OH-cortisol A_e_ (24-h-urine)
	μg*min/L	μg*min/L	ng/ml	μg/24 h
Mean	464.0	86.2	25.9	222
Variance within subjects (Var_w_)	4552	184	14.3	4247
Variance between subjects (Var_b_)	11233	2726	86.5	8141
Genetic component*	0.612	0.931	0.812^#^	0.457

*The genetic component was calculated from the variants between subjects (Var_b_) and within subjects (Var_w_) as Var_b_-Var_w_/Var_b_. That parameter reflects the maximum proportion of variation possibly attributable to genetic effects and/or constant environmental factors (e.g., 61.2% in case of midazolam AUC). ^#^Validity may be comprised by the fact that half-life of 4ß-OH-cholesterol is more than 60 h and thus with a minimum sampling interval of 168 h, possibly no completely independent sampling was obtained in some cases.

**TABLE 4:** Endogenous biomarkers of CYP3A activity.

Parameter	MZ	DZ
**Plasma**		
Cholesterol [mg/ml]	1.39 (0.86 – 1.99)	1.38 (0.94 – 2.12)
4ß-OH-cholesterol [ng/ml]	24.56 (12.59 – 62.96)	23.07 (15.12 – 46.77)
Ratio (cholesterol)	0.17 (0.01 – 0.35)	0.17 (0.12 – 0.25)
**Urine**		
Cortisol [μg]	35.85 (10.05 – 142.80)	32.14 (11.10 – 90.00)
6ß-OH-cortisol [μg]	209.0 (56.20 – 594.3)	193.9 (76.10 – 567.0)
Ratio (cortisol)	6.29 (3.09 – 14.65)	6.75 (3.38 – 18.22)

*Data was calculated from the mean of the up to three different study days of intravenous midazolam 0.2 mg administration and is given as median (minimum – maximum). Ratio (cholesterol) was calculated as 4ß-OH-Cholesterol / Cholesterol and expressed as (ng/ml)/(ng/ml) ^∗^ 10^4^. Cortisol and 6ß-OH-Cortisol are given as total amount collected in 24 h urine sampling. Ratio (cortisol) was calculated as 6ß-OH-Cortisol/Cortisol.*

**FIGURE 2:**
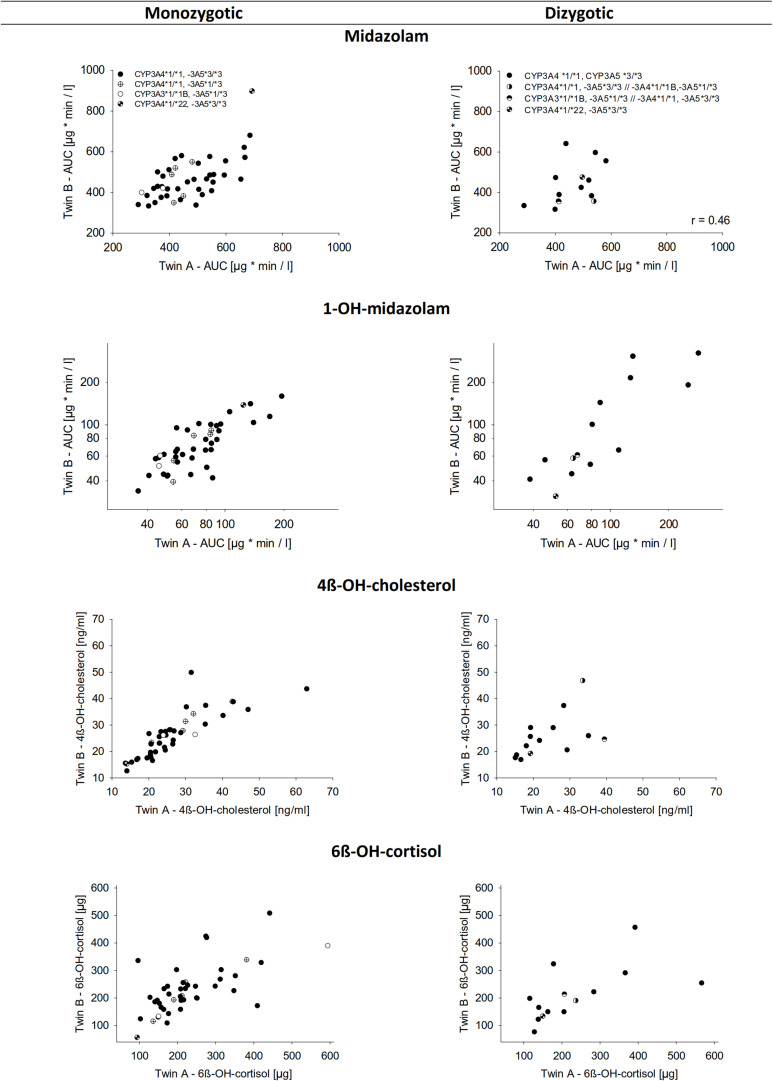
Correlation of the biomarkers for CYP3A activity between monozygotic and dizygotic twins. The upper lane shows midazolam AUC. Below is the AUC of 1-OH-midazolam, 4ß-OH-cholesterol in plasma, and 6ß-OH-cortisol in urine over 24 h. Data represents the mean of three measurements performed on separate days.

For all four indicators of CYP3A activity, i.e., midazolam, 1-OH-midazolam, 4ß-OH-cholesterol, and 6ß-OH-cortisol, there was a significant correlation between both, the MZ and the DZ pairs (Table 5 and Figure 2). A relatively high correlation of all 4 biomarkers even in the dizygotic twins might indicate that the respective parameters are modulated not only be heritable factors, but also by common environmental factors. But with all four biomarkers, the correlations between MZ twins were closer than between DZ twins, which supports the hypothesis that the biomarkers are also modulated by inherited effects (Table 5). Using structural equation analysis, based on the ACE model including additive genetic (A), common environment (C), and unique environmental (E) effects, the study showed a significant and high heritability only for 1-OH-midazolam (*p* = 0.03) and 4ß-hydroxy-cholesterol (*p* < 0.01) (Table 5, ACE columns). According to this data, 73% of the variation in 1-OH-midazolam and 56% of variation in plasma 4ß-OH-cholesterol were due to inherited factors. In contrast, variations of midazolam AUC and of 24-h urine 6ß-OH-cortisol were less strongly determined by genetic factors but more variation was explained by common environmental factors. For comparison, the two more parsimonious models are also given in Table 5, attributing all variation either to heritability (AE model) or to common environmental effects (CE model).

**TABLE 5:** Heritable and environmental effects on four known or presumed probe substrates of CYP3A activity.

Traits	Unstructured		ACE		AE		CE	
	*r*_mz_	*r*_dz_	*H*^2^	*C*^2^	*H*^2^	*C*^2^	*H*^2^	*C*^2^
Midazolam (AUC)	0.66 (0.06)*	0.52 (0.13)*	**0.15 (0.25)**	0.48 (0.24)*	0.63 (0.06)*	–	–	0.60 (0.06)*
1-OH-midazolam (AUC)	0.94 (0.01)*	0.59 (0.11)*	0.73 (0.23)*	0.21 (0.23)	0.94 (0.01)*	–	–	0.78 (0.04)*
4ß OH-cholesterol	0.79 (0.04)*	0.53 (0.12)*	0.56 (0.26)*	0.22 (0.26)	0.79 (0.04)*	–	–	0.74 (0.04)*
6ß OH-cortisol	0.68 (0.06)*	0.50 (0.13)*	**0.31 (0.27)**	0.36 (0.26)	0.68 (0.06)*	–	–	0.61 (0.06)*
LL(df)	−2280.1 (df = 36)		−2249.9 (df = 42)		−2253.0 (df = 32)		−2274.6 (df = 32)	
AIC	4632.3		4583.7		4570.1		4613.2	
BIC	4780.7		4756.8		4702.0		4745.1	

4ß OH-cholesterol is given as the morning plasma concentrations and 6ß OH-cortisol is given as the total 24 h urinary excretion (A_e_). In the left block, the correlations for identical (*r*_mz_) and fraternal (*r*_dz_) twins are given. Then the parameter of the ACE model with additive genetic effects (A), common environmental effects (C) and unique environmental effects (E) are provided as heritability (*H*^2^) and common environment (C^2^) effects and associated standard errors are given. Numbers highlighted in bold letters indicate the heritability according to structural equation modeling with A, C, and E effects. For comparison, also the more parsimonious models with additive genetic effects and unique environmental effects only (AE) and the model with common environmental effects and unique environmental effects are provided. Log-likelihood (LL) degrees of freedom (df), Akaike (AIC) and Bayesian (BIC) information criterion for the fitted models (bottom). *Significant to 95% of confidence.

### Correlation Between the Biomarkers of CY3A Activity

**FIGURE 3:**
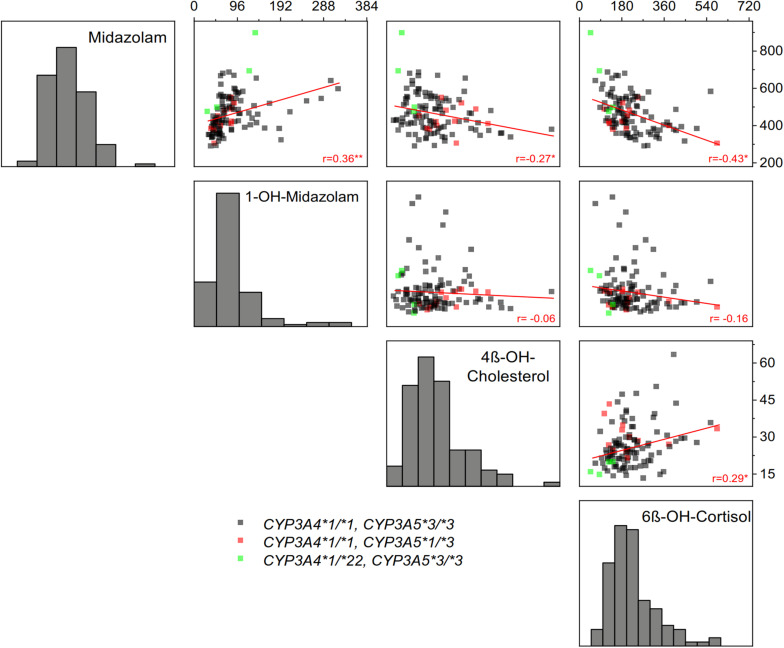
Correlations between the four indicators of CYP3A activity: plasma AUC of midazolam and 1-OH-midazolam, plasma concentration of 4ß-OH-cholesterol and the amount of 6ß-OH-cortisol excreted in 24-h urine. Linear regression coefficients are shown in the figure (*p < 0.05, **p < 0.01). As seen, there was a relatively high correlation between midazolam and 6ß-OH-cortisol, whereas the CYP3A generated metabolite 1-OH-midazolam did not correlate at all with the alternative endogenous *in vivo* biomarkers of CYP3A activity. Only frequent genetic polymorphisms with unequivocal functional relevance were considered, the CYP3A4*22 variant conferring low CYP3A4 activity and the CYP3A5*3 variant conferring loss of CYP3A5 activity.

As illustrated in Figure 3, correlations between the four biomarkers were surprisingly low or in some comparisons even absent. Notably, there was no correlation between 1-OH-midazolam and the two endogenous biomarkers of CYP3A. The simple phenotypic correlation as illustrated in Figure 3, does not indicate the origin of the correlation. However, in multivariate structural equation modeling (Table 6), one may employ either genetic and environmental correlations or phenotypically standardized covariances to assess the structure of the genetic and environmental influences. The genetic correlation is an estimate of the additive genetic effect that is shared between a pair of traits. It is obtained as a standardization of the additive genetic covariance between traits by their respective additive variances. As shown in Table 6, that parameter was statistically significant with coefficients of 0.4 only for midazolam and its metabolite (which may appear self-evident because of the drug-metabolite relationship), and, interestingly, for midazolam and 6ß-OH-cortisol, reassuring concerning the use of the latter two biomarkers as indicators of partially heritable CYP3A activity.

**TABLE 6:** Phenotypic correlation, genetic correlation, non-shared correlation, and bivariate heritability of 4 biomarkers of CYP3A activity.

	Midazolam	1-OH-midazolam	4ß-OH-cholesterol	6ß-OH-cortisol
**Phenotypic correlations (*r*_ph_)**				
Midazolam	1	0.37 (0.07)*	−0.24 (0.07)*	−0.41 (0.06)*
1-OH-midazolam		1	0.01 (0.08)	−0.13 (0.08)
4ß-OH-cholesterol			1	0.26 (0.07)*
6ß-OH-cortisol				1
**Genetic correlations (*r*_g_)**				
Midazolam	1	0.37 (0.09)*	−0.18 (0.11)	−0.42 (0.10)*
1-OH-midazolam		1	0.04 (0.09)	−0.15 (0.10)
4ß-OH-cholesterol			1	0.11 (0.11)
6ß-OH-cortisol				1
**Non-shared correlation (*r*_e_)**				
Midazolam	1	0.54 (0.08)*	−0.43 (0.09)*	−0.38 (0.09)*
1-OH-midazolam		1	−0.23 (0.10)*	−0.07 (0.11)
4ß-OH-cholesterol			1	0.69 (0.06)*
6ß-OH-cortisol				1
**Bivariate heritability**				
Midazolam	1	0.78 (0.07)*	0.52 (0.19)*	0.68 (0.11)*
1-OH-midazolam		1	3.19 (14.89)	0.92 (0.13)*
4ß-OH-cholesterol			1	0.30 (0.23)
6ß-OH-cortisol				1

Estimates (standard errors) * Significant to 95% of confidence. For midazolam and 1-OH midazolam, AUC was used in structural equation modeling as presented here.

### Variance Explained by Environmental Factors and Molecularly Defined Polymorphisms

Midazolam pharmacokinetics were significantly influenced by sex, lean body weight, type of nutrition, usage of hormonal contraceptives, and by the *CYP3A4^∗^22* polymorphism (Table 7). Median (minimum – maximum) midazolam AUC was 8% higher in male than in female subjects [466.0 μg ^∗^ min/L (290.6 – 668.1 μg ^∗^ min/L) versus 432.1 μg ^∗^ min/L (287.8 – 897.4 μg ^∗^ min/L)]. Higher lean body mass was associated with low AUC according to linear regression analysis. The median midazolam AUC in heterozygous carriers of the *CYP3A4^∗^22* polymorphism (*n* = 4) was 35% higher than in subjects with the *^∗^1/^∗^1* and *^∗^1/^∗^1B* genotypes (*n* = 110) (595.1 μg ^∗^ min/L versus 439.1 μg ^∗^ min/L).

**TABLE 7:** Genetic and environmental effects on the CYP3A probe drugs and biomarkers.

Biomarker	Midazolam (AUC)		1-OH-midazolam (AUC)		4ß-OH-cholesterol		6ß-OH-cortisol	
Factors	*r*^2^* (*p*)	Coefficient	*r*^2^ (*p*)	Coefficient	*r*^2^ (*p*)	Coefficient	*r*^2^ (*p*)	Coefficient
All	0.29 (<0.001)		0.15 (0.03)		0.24 (<0.001)		0.10 (0.02)	
Sex	0.03 (0.04)	74.51		11.79		−3.32		−9.28
Age		−0.60	0.05 (0.01)	−1.78		0.16		0.94
Lean body weight	0.08 (0.001)	−7.29		−1.74.		−0.20		−1.35
Smoking status		−21.79		1.46		3.28		−8.66
Oral contraceptives	0.05 (0.008)	−63.18	0.05 (0.02)	−31.33		−2.57		3.37
CYP3A4*22	0.08 (0.001)	169.57		−12.53		−8.21	0.05 (0.02)	−127.40
CYP3A5*3		32.33		29.27	0.04 (0.02)	−6.30		0.70

*The correlation is given for the model with all factors studied and below the partial correlation coefficients are given for significant factors only. For midazolam, coefficients indicate higher AUC in males, decrease with higher lean body weight, lower AUC with current use of contraceptives and higher AUC in carriers of CYP3A4*22. The *r*^2^ indicates the fraction of the total variation explained by all variables or by the respective factors. For 4ß-OH-cholesterol, coefficients indicate decrease of AUC per CYP3A5*3 allele. 6ß-OH-cortisol: Coefficients indicate decrease per CYP3A4*22 allele. The sample included no homozygous, but 4 heterozygous carriers of the CYP3A4*22 allele (3.4%) and no homozygous, but 16 heterozygous carriers of the CYP3A5*1 allele (13.8%).

The AUC of 1-OH-midazolam was significantly lower with increasing age (*p* = 0.012), but there were no statistically significant effects of the *CYP3A* genotypes (Table 7). Interestingly, plasma 4ß-OH-cholesterol was significantly lower in carriers of the *CYP3A5^∗^3* allele (*p* = 0.02), with a median 4ß-OH-cholesterol level of 23.01 ng/ml in homozygous carriers of the *CYP3A5^∗^3* allele and 30.69 ng/ml in heterozygous carriers of the active *CYP3A5^∗^1* allele. Our study included 16 carriers of the *CYP3A5^∗^1* genotype, and from the genotype-related differences (Table 3) one may conclude that *in vivo* CYP3A5 contributes in a relevant manner to cholesterol hydroxylation whereas CYP3A5 does not appear to contribute in a relevant manner to midazolam or cortisol hydroxylation. However, a minor contribution cannot be ruled out since our study was not designed to quantify the effects of specific *CYP3A4* or *CYP3A5* genetic variants.

Nutrition may affect CYP3A activity and expression. However, the analysis of nutritional effects was not a primary aim of the present study, and the data should thus be considered as explorative. High protein consumption was associated in a dose-dependent manner with low 1-OH-midazolam AUC and low 6ß-OH-cortisol. However, no effects of diet on 4ß-OH-cholesterol were found. High fruit intake was apparently associated with a high midazolam clearance, with a median AUC of 425.4, 446.9, 519.4, and 558.9 μg ^∗^ min/L in subjects with daily, thrice weekly, rare and very rare fruit consumption, respectively. Alcohol consumption may also modulate CYP3A activity but our data could not confirm that because, according to the standardized nutrition questionnaires, only three participants stated that they drank alcohol 1–3 times a week, and all the others drank less alcohol according to the questionnaires. According to alcohol breath tests and according to the liver function tests, there was also no indication of alcohol consumption.

## Discussion

Human CYP3A activity is highly variable between subjects. Some data published earlier indicated that up to 98% of that variation may be heritable (Ozdemir et al., 2000; Klein and Zanger, 2013). Up to 66% of induced CYP3A activity may be heritable, according to a twin study using a herbal product, St. John’s wort, for enzyme induction (Rahmioglu et al., 2011). However, there is scarce data from twin studies on heritability of not induced CYP3A activity. Because much of the current thinking about a high heritability in CYP3A activity is based on the high intra-individual constancy of CYP3A *in vivo* indicators, we designed our study to allow within the study a comparison between that repeated dose approach or *genetic component* approach (Ozdemir et al., 2000) and the twins study approach specifically reflecting heritabilty. As show in Table 3, there was significant intraindividual constancy but also some variation particularly in midazolam AUC (Figure 1C). Thus, between the study days environmental effects currently not classified as strong CYP3A inducers or inhibitors apparently may have had modified enzyme expression and activity and further research on such possibly factors (e.g., specific foods or changes in the intestinal microbiome) that may reveal interesting new insights. Our study participants were instructed to avoid any known inducer or inhibitor of CYP3A enzymes. Nevertheless, not yet identified inducers or inhibitors from nutrition, from the microbiome and from endogenous metabolism may have modulated CYP3A expression and activity in our study.

With a relatively small number of dizygotic twins, the differentiation between additive genetic effects (A) and constantly acting environmental (including epigenetic) effects (C) was not very precise as given in Table 5. But the sum of both, A and C (Table 5), was about 60% and that surprisingly well corresponded to the independent analytical approach based in intra- versus interindividual variation (Table 3).

A high intra-individual constancy (Figure 1D and Table 3) and a high heritability was indeed found for 1-OH-midazolam AUC (Table 5), but apparently this was due to a high heritability of glucuronyltransferases or other processes relevant for elimination of 1-OH-midazolam. In general, the low to moderate correlation between all four markers indicates that each biomarker also reflects processes beside CYP3A activity. The twin study design with multivariate structural equation analyses allowed exploring more about the causes. A low correlation of 4ß-OH-cholesterol with the other parameters may be due to the long half-life of 4ß-OH-cholesterol. Therefore, this biomarker does more reflect the overall integrated effects of CYP3A inducing and inhibiting factors over a longer time span, whereas the other three biomarkers better reflect the metabolic status at the day of testing. Indeed, the highest genetic correlation of −0.42 was found between midazolam and 6ß-OH-cortisol (Table 6) consistent with the assumption that both reflect a common genetic component in the CYP3A genes or in other genes regulating CYP3A genes.

Due to its high sensitivity to inhibitors and inducers, midazolam is currently a preferred CYP3A probe drug in drug research, but data shown here indicate that midazolam may also reflect other processes and activities besides CYP3A. With a clearance of about 0.5 L/min (Table 2), midazolam is a so-called high clearance drug (Foster et al., 2011; Burke et al., 2014) and all high-clearance drugs do depend in their elimination strongly on liver blood flow (Nies et al., 1976). However, the factors known to affect liver blood flow like physical activity or food intake, were highly controlled here on all three study days. Nevertheless, there was an apparently significant intra-individual variation in several volunteers between the three study days (Figure 1). With a mean coefficient of variation of 11.3% for intra-individual variation, the intra-individual variation in our study was overall not significantly higher than that observed in an earlier study also with repeated intravenous administration of midazolam (Kashuba et al., 1998). A moderately bigger intra-individual variation in our study may simply be due to the fact that in our study the time between the measurements was longer (on average 1 month). Ideally, time between measurements should not be relevant for truly only genetically determined factors, but the genetic component (Kalow et al., 1998) does not differentiate between A and C (additive genetic effects and constant environmental effects, Table 5). This intraindividual variation together with the common genetic correlation of only 0.42 between 6ß-OH-cortisol and midazolam indicates that there were significant random and individually constant effects modulating the midazolam pharmacokinetics in humans. Such constant environmental factors may include long-term epigenetic imprinting but also constant factors in lifestyle and nutrition. Subjects in our study were not allowed to consume known inducers or inhibitors of CYP3A like grapefruits or St. John’s wort. However, in an explorative analysis based on standardized nutrition questionnaires high fruit intake was associated with high midazolam clearance. This may support the hypothesis that there are CYP3A inducers in some fruits and vegetables, but the food questionnaire was not specific enough and sample size was not sufficient to analyze that in more detail. But we think that this is an interesting point of further research, possibly also including the interactions between CYP3A activity, food and the intestinal microbiome.

*In vitro*, one often prefers measuring enzyme activity based on the metabolite formation. *In vivo*, in our study, there was a high intra-individual constancy and significant heritability of the main midazolam metabolite 1-OH-midazolam (Figure 1 and Tables 3, 5). However, the 1-OH-midazolam AUC is determined by both metabolite formation and elimination. While metabolite formation is mostly mediated by CYP3A4, the AUC of 1-OH-midazolam was not different for the functionally relevant *CYP3A4^∗^22* polymorphisms and correlated only poorly with the other CYP3A biomarkers. This indicates that the variation in elimination of 1-OH-midazolam may be more important than the variation in its formation for our understanding of the 1-OH-midazolam plasma concentrations. That is also strongly supported by another finding: As seen in Figure 3, there was a positive correlation between midazolam and 1-OH-midazolam. With the substrate-product relationship between midazolam and 1-OH-midazolam that correlation should have been negative. This paradox is best explained by the assumption that plasma 1-OH-midazolam concentrations were much more dependent on factors other than CYP3A activity. That corresponds to earlier findings that under rifampin induction there was less plasma 1-OH-midazolam contrary to the expectations and only with analysis of the 1-OH-midazolam glucuronide there was more after rifampin induction (Link et al., 2008). Unfortunately, in our study the 1-OH-midazolam glucuronide was not analyzed. Elimination of 1-OH-midazolam may mostly be determined by glucuronidation via uridine 5’-diphospho-glucuronosyltransferase (UGT) enzymes 1A4, 2B6, and 2B7 (Seo et al., 2010; Stingl et al., 2014). The apparent high heritability in 1-OH-midazolam AUC may reflect, to a great extent, a high heritability of the corresponding UGT enzyme. Alternatively, membrane transporters relevant for 1-OH-midazolam and its glucuronide may be highly heritable. This remains speculative without further experimental identification, but at least it may teach us two points: firstly, drug-to-metabolite ratios may sometimes be misleading (or at least not increase specificity), and, secondly, highly predictive genetic polymorphisms may exist in the relevant UGT enzymes or in the glucuronide membrane transport proteins which should be further studied.

Our data on the poor correlation between 4ß-OH-cholesterol and midazolam AUC is in line with results published by others: For instance, the correlation between midazolam total clearance and 4ß-OH-cholesterol plasma concentration was only 0.24 (*p* = 0.048) in 50 healthy volunteers (Tomalik-Scharte et al., 2009). This is similar to the results obtained here (Figure 3). One explanation lies in the large differences in pharmacokinetics. While midazolam has an elimination half-life of about 2 h (Table 2), the half-life of 4ß-OH-cholesterol was estimated to be 60 h by application of radiolabeled substance to humans (Bodin et al., 2002) but other studies estimated that half-life even as 17 days or longer (Diczfalusy et al., 2008). Thus, 4ß-OH-cholesterol is an indicator for the mean average CYP3A activity over weeks whereas midazolam reflects CYP3A activity within a time span of a few hours only. There may exist moderate circadian variation in CYP3A activity, but this can only explain part of the missing correlation. Again, the pathways mediating further elimination of 4ß-OH-cholesterol by membrane transport and metabolism may play a role, and thus, the high heritability seen with 4ß-OH-cholesterol may not only be heritability related to CYP3A activity.

The cortisol metabolite 6ß-OH-cortisol has been in use in clinical pharmacology for more than 50 years (Roots et al., 1975). Under baseline conditions excluding any of the known strong inducers, there was only a moderate correlation between midazolam and 6ß-OH-cortisol in our study (Figure 3). Other studies found even no correlation at all between midazolam systemic clearance and the 6ß-OH-cortisol urinary ratio under such baseline conditions (Eeckhoudt et al., 2001; Chan et al., 2016). However, a high correlation was found between midazolam clearance and the 6ß-OH-cortisol ratio following hepatic CYP3A inhibition and induction (Shin et al., 2016). The endogenous plasma concentrations of 4ß-OH-cholesterol were similarly identified as a suitable biomarker correlating with midazolam after long-term CYP3A induction (Kasichayanula et al., 2014). However, with enzyme induction, probably all four *in vivo* markers of CYP3A activity used here most likely would have correlated much stronger than found here. Heritability of induced CYP3A activity was indeed found to be about 66% after a 14 days treatment with St. John’s wort (Rahmioglu et al., 2011), but the heritability of baseline (not significantly induced) CYP3A activity was not studied there.

## Conclusion

In a population not exposed to known inducers or inhibitors, heritability of CYP3A activity was found to be relatively small, particularly when using midazolam as biomarker. Considering the known very strong susceptibility of CYP3A4 and CYP3A5 to enzyme induction and enzyme inhibition, this finding may not appear unexpected. In our study, the estimates of CYP3A heritability varied depending on the biomarker used, which could partially be explained by the specific features of each probe substance, but it clearly indicates that the one and only correct *in vivo* biomarker of CYP3A activity does not exist. The multivariate twin study design allowed searching for the common hidden factor using structural equation modeling, but this did not identify a strong genetic correlation between all four presumed CYP3A biomarkers. The relatively poor correlation between different biomarkers of CYP3A activity may not generally devalue them, but we should keep in mind that CYP3A biomarkers may reflect to a significant extent more of other processes than commonly presumed. The fraction of variation in CYP3A4 activity currently explained by defined genetic polymorphisms is relatively small and according to our study, search for further genomic biomarkers to predict reliably the individual CYP3A activity may not be very promising. Instead, functional CYP3A biomarkers may be more suitable for therapy individualization and further search for epigenetic factors modulating intermediate- and long-term CYP3A activity may be promising. However, the high heritability of 1-OH-midazolam might stimulate further research on the underlying genetic polymorphisms relevant for this metabolite.

## Data Availability Statement

The raw data supporting the conclusions of this article will be made available by the authors, without undue reservation.

## Ethics Statement

The studies involving human participants were reviewed and approved by the Ethikkommission der Universitätsmedizin Göttingen, Georg-August-Universität Göttingen. The patients/participants provided their written informed consent to participate in this study.

## Author Contributions

JM, WB, and JB wrote the manuscript. JB, DS, MS, and RK designed the research. JM, JB, MT, JS, DS, CS-S, and UH performed the research. WB, JB, JM, and JB analyzed the data. All authors contributed to the article and approved the submitted version.

## Conflict of Interest

The authors declare that the research was conducted in the absence of any commercial or financial relationships that could be construed as a potential conflict of interest.
